# Heterogeneous Graphical Granger Causality by Minimum Message Length

**DOI:** 10.3390/e22121400

**Published:** 2020-12-11

**Authors:** Kateřina Hlaváčková-Schindler, Claudia Plant

**Affiliations:** 1 Faculty of Computer Science, University of Vienna, 1090 Wien, Austria; claudia.plant@univie.ac.at; 2 Institute of Computer Science of the Czech Academy of Sciences, 18207 Prague, Czech Republic; 3 ds:UniVie, University of Vienna, 1090 Wien, Austria

**Keywords:** Granger causality, graphical Granger model, overestimation, information theory, minimum message length

## Abstract

The heterogeneous graphical Granger model (HGGM) for causal inference among processes with distributions from an exponential family is efficient in scenarios when the number of time observations is much greater than the number of time series, normally by several orders of magnitude. However, in the case of “short” time series, the inference in HGGM often suffers from overestimation. To remedy this, we use the minimum message length principle (MML) to determinate the causal connections in the HGGM. The minimum message length as a Bayesian information-theoretic method for statistical model selection applies Occam’s razor in the following way: even when models are equal in their measure of fit-accuracy to the observed data, the one generating the most concise explanation of data is more likely to be correct. Based on the dispersion coefficient of the target time series and on the initial maximum likelihood estimates of the regression coefficients, we propose a minimum message length criterion to select the subset of causally connected time series with each target time series and derive its form for various exponential distributions. We propose two algorithms—the genetic-type algorithm (HMMLGA) and exHMML to find the subset. We demonstrated the superiority of both algorithms in synthetic experiments with respect to the comparison methods Lingam, HGGM and statistical framework Granger causality (SFGC). In the real data experiments, we used the methods to discriminate between pregnancy and labor phase using electrohysterogram data of Islandic mothers from Physionet databasis. We further analysed the Austrian climatological time measurements and their temporal interactions in rain and sunny days scenarios. In both experiments, the results of HMMLGA had the most realistic interpretation with respect to the comparison methods. We provide our code in Matlab. To our best knowledge, this is the first work using the MML principle for causal inference in HGGM.

## 1. Introduction

Granger causality is a popular method for causality analysis in time series due to its computational simplicity. Its application to time series with non-Gaussian distributions can be, however, misleading. Recently, Behzadi et al. in [1] proposed the heterogeneous graphical Granger Model (HGGM) for detecting causal relations among time series with distributions from the exponential family, which includes a wider class of common distributions. HGGM employs regression in generalized linear models (GLM) with adaptive Lasso penalization [2] as a variable selection method and applies it to time series with a given lag. This approach allows one to apply causal inference among time series, also with discrete values. HGGM, using penalization by adaptive Lasso, showed its efficiency in scenarios when the number of time observations is much greater than the number of time series, normally by several orders of magnitude—however, on “short” time series, the inference in HGGM suffers often from overestimation.

Overestimation on short time series is a problem which also occurs in general forecasting problems. For example, when forecasting demand for a new product or a new customer, there are usually very few time series observations available. For such short time series, the traditional forecasting methods may be inaccurate. To overcome this problem in forecasting, Ref. [3] proposed to utilize a prior information derived from the data and applied a Bayesian inference approach. Similarly for another data mining problem, a Bayesian approach has shown to be efficient for the clustering of short time series [4].

Motivated by the efficiency of the Bayes approaches in these problems on short time series, we propose to use the Bayesian approach called minimum message principle, as introduced in [5] to causal inference in HGGM. The contributions of our paper are the following:

(1) We used the minimum message length (MML) principle for determination of causal connections in the heterogeneous graphical Granger model.
(2) Based on the dispersion coefficient of the target time series and on the initial maximum likelihood estimates of the regression coefficients, we proposed a minimum message length criterion to select the subset of causally connected time series with each target time series; Furthermore, we derived its form for various exponential distributions.
(3) We found this subset in two ways: by a proposed genetic-type algorithm (HMMLGA), as well as by exhaustive search (exHMML). We evaluated the complexities of these algorithms and provided the code in Matlab.
(4) We demonstrated the superiority of both methods with respect to the comparison methods Lingam [6], HGGM [1] and statistical framework Granger causality (SFGC) [7] in the synthetic experiments with short time series. In the real data experiments without known ground truth, the interpretation of causal connections achieved by HMMLGA was the most realistic with respect to the comparison methods.
(5) To our best knowledge, this is the first work applying the minimum message length principle to the heterogeneous graphical Granger model.

The paper is organized as follows. Section 2 presents definitions of the graphical Granger causal model and of the heterogeneous graphical Granger causal model as well as of the minimum message length principle. Our method including the derived criteria and algorithm are described in Section 3. Related work is discussed in Section 4. Our experiments are summarized in Section 5. Section 6 is devoted to the conclusions and the derivation of the criteria from Section 3 can be found in Appendix A and Appendix B.

## 2. Preliminaries

To make this paper self-contained and to introduce the notation, we briefly summarize the basics about graphical Granger causal model in Section 2.1. The heterogeneous graphical Granger model, as introduced in [1], is presented in Section 2.2. Section 2.3 discusses the strengths and limitations of the Granger causal models. The idea of the minimum message length principle is briefly explained in Section 2.4.

### 2.1. Graphical Granger Model

The (Gaussian) graphical Granger model extends the autoregressive concept of Granger causality to p≥2 time series [8]. Let $x_{1}^{t},\ldots ,x_{p}^{t}$ be the time instances of *p* time series, t=1,…,n. As it is common, we will use bold font in notation of vectors or matrices. Consider the vector auto-regressive (VAR) models with time lag d≥1 for i=1,…,p

(1) $$x_{i}^{t}=X_{t,d}^{Lag}\beta_{i}^{\prime }+\varepsilon_{i}^{t}$$

where $X_{t,d}^{Lag}=\left(x_{1}^{t-d},\ldots ,x_{1}^{t-1},\ldots ,x_{p}^{t-d},\ldots ,x_{p}^{t-1}\right)$ and $\beta_{i}$ be a matrix of the regression coefficients and $\varepsilon_{i}^{t}$ be white noise. One can easily show that $X_{t,d}^{Lag}\beta_{i}^{\prime }=\sum_{j=1}^{p}\sum_{l=1}^{d}x_{j}^{t-l}\beta_{j}^{l}$.

**Definition** **1.** *One says time series $x_{j}$ Granger–causes time series $x_{i}$ for a given lag d, denote $x_{j}\to x_{i}$, for i,j=1,…,p if and only if at least one of the d coefficients in j-th row of $\beta_{i}$ in (1) is non-zero.*

The solution of problem (1) has been approached by various forms of penalization methods in the literature, e.g., Lasso in [8], truncated Lasso in [9] or group Lasso [10].

### 2.2. Heterogeneous Graphical Granger Model

The heterogeneous graphical Granger model (HGGM) [1] considers time series $x_{i}$, for which their likelihood function belongs into the exponential family with a canonical parameter $\theta_{i}$. The generic density form for each $x_{i}$ can be written as

(2) $$p\left(x_{i}|X_{t,d}^{Lag},\theta_{i}\right)=h\left(x_{i}\right)\exp\left(x_{i}\theta_{i}-\eta_{i}\left(\theta_{i}\right)\right)$$

where $\theta_{i}=X_{t,d}^{Lag}{\left(\beta_{i}^{*}\right)}^{\prime }$ ($\beta_{i}^{*}$ is the optimum) and $\eta_{i}$ is a link function corresponding to time series $x_{i}$. (The sign ${}^{\prime }$ denotes a transpose of a matrix). The heterogeneous graphical Granger model uses the idea of generalized linear models (GLM, see e.g., [11]) and applies them to time series in the following form

(3) $$x_{i}^{t}\approx \mu_{i}^{t}=\eta_{i}^{t}\left(X_{t,d}^{Lag}\beta_{i}^{\prime }\right)=\eta_{i}^{t}\left(\sum_{j=1}^{p}\sum_{l=1}^{d}x_{j}^{t-l}\beta_{j}^{l}\right)$$

for $x_{i}^{t}$, i=1,…,p,t=d+1,…,n each having a probability density from the exponential family; $\mu_{i}$ denotes the mean of $x_{i}$ and $var\left(x_{i}|\mu_{i},\phi_{i}\right)=\phi_{i}v_{i}\left(\mu_{i}\right)$ where $\phi_{i}$ is a dispersion parameter and $v_{i}$ is a variance function dependent only on $\mu_{i}$; $\eta_{i}^{t}$ is the *t*-th coordinate of $\eta_{i}$.

Causal inference in (3) can be solved as

(4) $${\hat{\beta }}_{i}=arg\min_{\beta_{i}}\sum_{t=d+1}^{n}\left(-x_{i}^{t}\left(X_{t,d}^{Lag}\beta_{i}^{\prime }\right)+\eta_{i}^{t}\left(X_{t,d}^{Lag}\beta_{i}^{\prime }\right)\right)+\lambda_{i}R\left(\beta_{i}\right)$$

for a given lag d>0, $\lambda_{i}>0$, and all t=d+1,…,n with $R(\beta_{i})$ to be the adaptive Lasso penalty function [1]. (The first two summands in (4) correspond to the maximum likelihood estimates in the GLM).

**Definition** **2.** *One says, time series $x_{j}$ Granger–causes time series $x_{i}$ for a given lag d, denote $x_{j}\to x_{i}$, for i,j=1,…,p if and only if at least one of the d coefficients in j-th row of $\hat{\beta_{i}}$ of the solution of (4) is non-zero* [1].

**Remark** **1.** *Non-zero values in Definitions 1 and 2 are in practice, distinguished by considering values bigger than a given threshold, which is a positive number “close” to zero.*

For example, Equation (4) for the Poisson graphical Granger model [12] where for each i=1,…,p$\eta_{i}^{t}:=\exp$ is considered, can be written as

(5) $${\hat{\beta }}_{i}=arg\min_{\beta_{i}}\sum_{t=d+1}^{n}\left(-x_{i}^{t}\left(X_{t,d}^{Lag}\beta_{i}^{\prime }\right)+\exp\left(X_{t,d}^{Lag}\beta_{i}^{\prime }\right)\right)+\lambda_{i}R\left(\beta_{i}\right).$$

Equation (4) for the binomial graphical Granger model can be written as

(6) $${\hat{\beta }}_{i}=arg\min_{\beta_{i}}\sum_{t=d+1}^{n}\left(-x_{i}^{t}\left(X_{t,d}^{Lag}\beta_{i}^{\prime }\right)+\log\left(1+\exp\left(X_{t,d}^{Lag}\beta_{i}^{\prime }\right)\right)\right)+\lambda_{i}R\left(\beta_{i}\right)$$

and finally Equation (4) for the Gaussian graphical Granger model reduces to the least squares error of (1) with a $R(\beta_{i})$ to be adaptive Lasso penalty function. The heterogeneous graphical Granger model can be applied to causal inference among processes, for example in climatology, e.g., Ref. [1] investigated the causal inference among precipitation time series (having gamma distribution) and time series of sunny days (having Poisson distribution).

### 2.3. Granger Causality and Graphical Granger Models

Since its introduction, Granger causality [13] has faced criticism, since it e.g., does not take into account counterfactuals, [14,15]. In defense of his method, Granger in [16] wrote: “Possible causation is not considered for any arbitrarily selected group of variables, but only for variables for which the researcher has some prior belief that causation is, in some sense, likely.” In other words, drawing conclusions about the existence of a causal relation between time series and about its direction is possible only if theoretical knowledge of mechanisms connecting the time series is accessible.

Concerning the graphical causal models, including the Granger ones, Lindquist and Sobel in [17] claim that (1) they are not able to discover causal effects; (2) the theory of graphical causal models developed by Spirtes et al. in [18] makes no counterfactual claims; and (3) causal relations cannot be determined non-experimentally from samples that are a combination of systems with different propensities. However, Glymour in [19] argues that each of these claims are false or exaggerated. For arguments against (1) and (3), we refer the reader to [19]. We focus here only to his arguments to (2). Quoting Glymour, claims about what the outcome would be of a hypothetical experiment that has not been done are one form of counterfactual claims. Such claims say that if such and such were to happen then the result would be thus and so—where such and such has not happened or has not yet happened. (Of course, if the experiment is later done, then the proposition becomes factually true or factually false.) Glymour argues that it is not true that the graphical model framework does not represent or entail any counterfactual claims and emphasizes that no counterfactual variables are used or needed in the graphical causal model framework. In the potential outcomes framework, if nothing is known about which of many variables are causes of the others, then for each variable, and for each value of the other variables, a new counterfactual variable is required. In practice that would require an astronomical number of counterfactual variables for even a few actual variables. To summarize, as also confirmed by a recent *Nature* publication [20], if the theoretical background of investigated processes is insufficient, graphical causal methods (Granger causality including), to infer causal relations from data rather than knowledge of mechanisms, are helpful.

### 2.4. Minimum Message Length Principle

The minimum message length principle of statistical and inductive inference and machine learning was developed by C.S. Wallace and D.M. Boulton in 1968 in the seminal paper [5]. Minimum message length principle is a formal information theory restatement of Occam’s razor: even when models are not equal in goodness of fit accuracy to the observed data, the one generating the shortest overall message is more likely to be correct (where the message consists of a statement of the model, followed by a statement of data encoded concisely using that model). The MML principle selects the model which most compresses the data (i.e., the one with the “shortest message length”) as the most descriptive for the data. To be able to decompress this representation of the data, the details of the statistical model used to encode the data must also be part of the compressed data string. The calculation of the exact message is an NP hard problem, however the most widely used less computationally intensive is the Wallace–Freeman approximation called MML87 [21]. MML is Bayesian (i.e., it incorporates prior beliefs) and information-theoretic. It has the desirable properties of statistical invariance (i.e., the inference transforms with a re-parametrisation), statistical consistency (i.e., even for very hard problems, MML will converge to any underlying model) and efficiency (i.e., the MML model will converge to any true underlying model about as quickly as is possible). Wallace and Dowe (1999) showed in [22] a formal connection between MML and Kolmogorov complexity, i.e., the length of a shortest computer program that produces the object as output.

## 3. Method

In this section, we will describe our method in detail. First, in Section 3.1, we will derive a fixed design matrix for HGGM, so that the minimum message length principle can be applied. In Section 3.2, we propose our minimum message length criterion for HGGM. The exact forms of the criterion for various exponential distributions are derived in Section 3.3. Then, we present our two variable selection algorithms and their computational complexity in Section 3.4 and Section 3.5.

### 3.1. Heterogeneous Graphical Granger Model with Fixed Design Matrix

We can see that the models from Section 2 do not have fixed matrices. Since the MML principle proposed for generalized linear models in [23] requires a fixed design matrix, it cannot be directly applied to them. In the following section, we will derive the heterogeneous graphical Granger model (3) with a fixed lag *d* as an instance of regression in generalized linear models (GLM) with a fixed design matrix.

Consider the full model for *p* variables $x_{i}^{t}$ and lag d≥1 (be an integer) corresponding to the optimization problem (3). To be able to use the maximum likelihood (ML) estimation over the regression parameters, we reformulate the matrix of lagged time series $X_{t,d}^{Lag}$ from (1) into a fixed design matrix form. Assume n−d>pd and denote $x_{i}=\left(x_{i}^{d+1},x_{i}^{d+2},\ldots ,x_{i}^{n}\right)$. We construct the (n−d)×(d×p) design matrix

(7) $$X=\left[\begin{array}{ccccccc} x_{1}^{d} & \ldots & x_{1}^{1} & \ldots & x_{p}^{d} & \ldots & x_{p}^{1}\\ x_{1}^{d+1} & \ldots & x_{1}^{2} & \ldots & x_{p}^{d+1} & \ldots & x_{p}^{2}\\ \vdots & \vdots & \vdots & \vdots & \vdots & \vdots & \vdots \\ x_{1}^{n-1} & \ldots & x_{1}^{n-d+1} & \ldots & x_{p}^{n-1} & \ldots & x_{p}^{n-d+1} \end{array}\right]$$

and the 1×(d×p) vector $\beta_{i}=\left(\beta_{1}^{1},\ldots ,\beta_{1}^{d},\ldots ,\beta_{p}^{1},\ldots ,\beta_{p}^{d}\right)$. We can see that problem

(8) $$x_{i}^{\prime }\approx \mu_{i}=\eta_{i}\left(X\beta_{i}^{\prime }\right)$$

for i=d+1,…,n is equivalent to problem (3) in the matrix form where $\mu_{i}=\left(\mu_{i}^{d+1},\ldots ,\mu_{i}^{d+1}\right)$ and link function $\eta_{i}$ operates on each coordinate.

Denote now by $\gamma_{i}\subset \Gamma =\left\{1,\ldots ,p\right\}$ the subset of indices of regressor’s variables and $k_{i}:=\left|\gamma_{i}\right|$ its cardinality. Let $\beta_{i}:=\beta_{i}\left(\gamma_{i}\right)\in R^{1\;\times \;(d\;\times \;k_{i})}$ be the vector of unknown regression coefficients with a fixed ordering within the $\gamma_{i}$ subset. For illustration purposes and without lack of generality, we can assume that the first $k_{i}$ indices out of *p* vectors belong to $\gamma_{i}$. Considering only the columns from matrix X in (7), which correspond to $\gamma_{i}$, we define the $\left(n-d\right)\;\times \;\left(d\;\times \;k_{i}\right)$ matrix of lagged vectors with indices from $\gamma_{i}$ as

(9) $$X_{i}:=X\left(\gamma_{i}\right)=\left[\begin{array}{cccccccc} x_{1}^{d} & \ldots & x_{1}^{1} & \ldots & x_{k_{i}}^{d} & x_{k_{i}}^{d-1} & \ldots & x_{k_{i}}^{1}\\ x_{1}^{d+1} & \ldots & x_{1}^{2} & \ldots & x_{k_{i}}^{d+1} & x_{k_{i}}^{d} & \ldots & x_{k_{i}}^{2}\\ x_{1}^{d+2} & \ldots & x_{1}^{3} & \ldots & x_{k_{i}}^{d+2} & x_{k_{i}}^{d+1} & \ldots & x_{k_{i}}^{3}\\ \vdots & \vdots & \vdots & \vdots & \vdots & \vdots & \vdots & \vdots \\ x_{1}^{n-1} & \ldots & x_{1}^{n-d+1} & \ldots & x_{k_{i}}^{n-1} & x_{k_{i}}^{n-2} & \ldots & x_{k_{i}}^{n-d+1} \end{array}\right]$$

The problem (8) for explanatory variables with indices from $\gamma_{i}$ is expressed as

(10) $$x_{i}^{\prime }\approx \mu_{i}=E\left(x_{i}^{\prime }|X_{i}\right)=\eta_{i}\left(X_{i}\beta_{i}^{\prime }\right).$$

with $\beta_{i}:=\beta_{i}\left(\gamma_{i}\right)$ to be a $1\;\times \;(dk_{i})$ matrix of unknown coefficients and $\eta_{i}$ operates on each coordinate. Wherever it is clear from context, we will simplify the notation $\beta_{i}$ instead of $\beta_{i}\left(\gamma_{i}\right)$ and $X_{i}$ instead of $X(\gamma_{i})$.

### 3.2. Minimum Message Length Criterion for Heterogeneous Graphical Granger Model

As before, we assume for each $x_{i}^{t}$, where i=1,…,p, t=d+1,…,n to have a density from the exponential family; furthermore, $\mu_{i}$ to be the mean of $x_{i}$ and $var\left(x_{i}|\mu_{i},\phi_{i}\right)=\phi_{i}v_{i}\left(\mu_{i}\right)$ where $\phi_{i}$ is a dispersion parameter and $v_{i}$ a variance function dependent only on $\mu_{i}$. In the concrete case, for Poisson regression, it is well known that it can be still used in over- or underdispersed settings. However, the standard error for Poisson regression would not be correct for the overdispersed situation. In the Poisson graphical Granger model, it is the case when, for the dispersion of at least one time series holds $\phi_{i}\neq 1$. In the following, we assume that an estimate of $\phi_{i}$ is given. Denote Γ the set of all subsets of covariates $x_{i},i=1,\ldots ,p$. Assume now a fixed set $\gamma_{i}\in \Gamma$ of covariates with size $k_{i}\leq p$ and the corresponding design matrix $X_{i}$ from (9). Furthermore, we assume that the targets $x_{i}$ are independent random variables, conditioned on the features given by $X_{i}$, so that the likelihood function can be factorized into the product $p\left(x_{i}|\beta_{i},X_{i},\gamma_{i}\right)=\prod_{t=1}^{n-d}p\left(x_{i}^{t}|\beta_{i},X_{i},\gamma_{i}\right)$. The log-likelihood function $L_{i}$ has then the form $L_{i}:=\log p\left(x_{i}|\beta_{i},X_{i},\gamma_{i}\right)=\sum_{t=1}^{n-d}\log p\left(x_{i}^{t}|\beta_{i},X_{i},\gamma_{i}\right).$ Since $X_{i}$ is highly collinear, to make the ill-posed problem for coefficients $\beta_{i}$ (8) a well-posed one, we could use regularization by the ridge regression for GLM (see e.g., [24]). Ridge regression requires an initial estimate of $\beta_{i}$, which can be set as the maximum likelihood estimator of (10) obtained by the iteratively reweighted least square algorithm (IRLS). For a fixed $\lambda_{i}>0$, for the ridge estimates of coefficients ${\hat{\beta }}_{i,\lambda_{i}}$ holds

(11) $${\hat{\beta }}_{i,\lambda_{i}}={arg min}_{\beta_{i}\in R^{1\;\times \;dk_{i}}}\left\{-L_{i}+\lambda_{i}\beta_{i}^{\prime }\Sigma_{i}\beta_{i}\right\}.$$

In our paper however, we will not use the GLM ridge regression in form (11), but we apply the principle of minimum description length. Ridge regression in the minimum description length framework is equivalent to allowing the prior distribution to depend on a hyperparameter (= the ridge regularization parameter). To compute the message length of HGGM using the MML87 approximation, we need the negative log-likelihood function, prior distribution over the parameters and an appropriate Fisher information matrix, similarly as proposed in [23], where it is done for a general GLM regression. Moreover, [23] proposed the corrected form of Fisher information matrix for a GLM regression with ridge penalty. In our work, we will use this form of ridge regression and apply it to the heterogeneous graphical Granger model. In the following, we will construct the MML code for every subset of covariates in HGGM. The derivation of the criterion can be found in Appendix A.

**The MML criterion for inference in HGGM.** *Assume $x_{i},i=1,\ldots ,p$ be given time series of length n having distributions from exponential family, and for each of them, the estimate of the dispersion parameter ${\hat{\phi }}_{i}$ is given. Consider ${\hat{\beta }}_{i}$ be an initial solution of (8) with a fixed d≥1 achieved as the maximum likelihood estimate. Then*

*(i)*  *the causal graph of the heterogeneous graphical Granger problem (8) can be inferred from the solutions of p variable selection problems, where for each i=1,…,p, the set ${\hat{\gamma }}_{i}$ of Granger–causal variables to $x_{i}$ is found;*
*(ii)*  *For the estimated set ${\hat{\gamma }}_{i}$ holds*
  (12) $$\hat{\gamma_{i}}=\arg\min_{\gamma_{i}\in \Gamma }\left\{HMML\left(x_{i},X_{i},\gamma_{i}\right)\right\}=\arg\min_{\gamma_{i}\in \Gamma }\left\{I\left(x_{i},{\hat{\beta }}_{i},{\hat{\phi }}_{i},{\hat{\lambda }}_{i},X_{i},\gamma_{i}\right)+I\left(\gamma_{i}\right)\right\}$$
*where $I\left(x_{i},{\hat{\beta }}_{i},{\hat{\phi }}_{i},\hat{\lambda_{i}},X_{i},\gamma_{i}\right)=\min_{\lambda_{i}\in R^{+}}\left\{MML\left(x_{i},{\hat{\beta }}_{i},{\hat{\phi }}_{i},\lambda_{i},X_{i},\gamma_{i}\right)\right\}$ and*
*$MML(x_{i},{\hat{\beta }}_{i},{\hat{\phi }}_{i},\lambda_{i},X_{i},\gamma_{i})$ is the minimum message length code of set $\gamma_{i}$. It can be expressed as*
  (13) $$MML\left(x_{i},{\hat{\beta }}_{i},{\hat{\phi }}_{i},\lambda_{i},X_{i},\gamma_{i}\right)=-L_{i}+\frac{1}{2}\log\det\left(X_{i}^{\prime }W_{i}X_{i}+\lambda_{i}\Sigma_{i}\right)$$
*$+\frac{k_{i}}{2}\log\left(\frac{2\pi }{\lambda_{i}}\right)$$+\left(\frac{\lambda_{i}}{2{\hat{\phi }}_{i}}\right){\hat{\beta }}_{i}^{\prime }\Sigma_{i}{\hat{\beta }}_{i}$$+\frac{1}{2}\log\left(n-d\right)$$-\frac{k_{i}+1}{2}\log\left(2\pi \right)$$+\frac{1}{2}\log\left(\left(k_{i}+1\right)\pi \right)$ where $|{\hat{\gamma }}_{i}|=k_{i}$, $\Sigma_{i}$ is the unity matrix of size $dk_{i}\;\times \;dk_{i}$, I(γi)=logpki+log(p+1), $L_{i}$ is the log-likelihood function depending on the density function of $x_{i}$ and matrix $W_{i}$ is a diagonal matrix depending on link function $\eta_{i}$.*

**Remark** **2.** ([23]) *compared $AIC_{c}$ criterion with MML code for generalized linear models. We constructed the $AIC_{c}$ criterion also for HGGM. This criterion however requires the computation of pseudoinverse of a matrix multiplication, which includes matrices $X_{i}$. Since $X_{i}$s are highly collinear, these matrix multiplications had, in our experiments, very high condition numbers. This consequently led to the application of $AIC_{c}$ for HGGM, giving spurious results, and therefore we do not report them in our paper.*

### 3.3. Log-Likelihood $L_{i}$, Matrix $W_{i}$ and Dispersion $\phi_{i}$ for $x_{i}$ with Various Exponential Distributions

In this section, we will present the form for the log-likelihood function and for matrix $W_{i}$ for Gaussian, binomial, Poisson, gamma and inverse-Gaussian distributed time series $x_{i}$. The derivation for each case can be found in Appendix B. $\mu_{i}=\eta_{i}\left(X_{i}\beta_{i}^{\prime }\right)$ holds in each case for the link function as in (10). By ${\left[X_{i}\beta_{i}^{\prime }\right]}^{t}$, we denote the *t*-th coordinate of vector $X_{i}\beta_{i}^{\prime }$.

**Case $x_{i}$ is Gaussian** This is the case when $x_{i}$ is an independent Gaussian random variable and link function $\eta_{i}$ is identity. Assume ${\hat{\phi }}_{i}=\sigma_{i}^{2}$ to be the variance of the Gaussian random variable. We assume that in model (10) $x_{i}$ follows Gaussian distribution with the density function $p(x_{i}|{\hat{\beta }}_{i},\sigma_{i}^{2},X_{i},\gamma_{i})=$

(14) $$\prod_{t=d+1}^{n}p\left(x_{i}^{t}|{\hat{\beta }}_{i},\sigma_{i}^{2},X_{i},\gamma_{i}\right)={\left(\frac{1}{\left(2\pi \sigma_{i}^{2}\right)}\right)}^{(n-d)/2}\exp\left[-\frac{1}{2\sigma_{i}^{2}}\sum_{t=d+1}^{n}{\left(x_{i}^{t}-{\left[X_{i}{\hat{\beta }}_{i}\right]}^{t}\right)}^{2}\right].$$

Then

(15) $$L_{i}=\log p\left(x_{i}|{\hat{\beta }}_{i},\sigma_{i}^{2},X_{i},\gamma_{i}\right)=-\frac{n-d}{2}\log\left(2\pi \sigma_{i}^{2}\right)-\frac{1}{2\sigma_{i}^{2}}\sum_{t=d+1}^{n}{\left(x_{i}^{t}-{\left[X_{i}{\hat{\beta }}_{i}\right]}^{t}\right)}^{2}$$

and $W_{i}:=I_{n-d,n-d}$ is a unit matrix of dimension (n−d)×(n−d).

**Case $x_{i}$ is binomial** This is the case when $x_{i}$ is an independent Bernoulli random variable and it can achieve only two different values. For the link function, it holds $\eta_{i}=\log\left(\frac{\mu_{i}}{1-\mu_{j}}\right)$. Without lack of generality, we consider ${\hat{\phi }}_{i}=1$ and the density function $p(x_{i}|{\hat{\beta }}_{i},\sigma_{i}^{2},X_{i},\gamma_{i})=$

(16) $$\prod_{t=d+1}^{n}p\left(x_{i}^{t}|{\hat{\beta }}_{i},\sigma_{i}^{2},X_{i},\gamma_{i}\right)=\prod_{t=d+1}^{n}{\left({\left[X_{i}{\hat{\beta }}_{i}^{\prime }\right]}^{t}\right)}^{x_{i}^{t}}{\left(1-\left({\left[X_{i}{\hat{\beta }}_{i}^{\prime }\right]}^{t}\right)\right)}^{\left(1-x_{i}^{t}\right)}.$$

Then

(17) $$L_{i}=\log\left(p\left(x_{i}|{\hat{\beta }}_{i},X_{i},\gamma_{i}\right)\right)=\sum_{t=d+1}^{n}\left(x_{i}^{t}{\left[X_{i}{\hat{\beta }}_{i}^{\prime }\right]}^{t}-\log\left(1+\exp{\left[X_{i}{\hat{\beta }}_{i}^{\prime }\right]}^{t}\right)\right)$$

and

(18) $$W_{i}:=\mathrm{diag}\left(\frac{\exp\left({\left[X_{i}{\hat{\beta }}_{i}\right]}^{1}\right)}{{\left(1+\exp\left({\left[X_{i}{\hat{\beta }}_{i}\right]}^{1}\right)\right)}^{2}},\ldots ,\frac{\exp\left({\left[X_{i}{\hat{\beta }}_{i}\right]}^{n-d}\right)}{{\left(1+\exp({\left[X_{i}{\hat{\beta }}_{i}\right]}^{n-d}\right)}^{2})}\right).$$

In the case that we cannot assume accurate fitting to one of the two values, for robust estimation we can consider the sandwich estimate of the covariance matrix of ${\hat{\beta }}_{i}$ with

(19) $$W_{i}=\mathrm{diag}\left({\left[x_{i}^{1}-\frac{\exp\left({\left[X_{i}{\hat{\beta }}_{i}^{\prime }\right]}^{1}\right)}{{\left(1+\exp\left({\left[X_{i}{\hat{\beta }}_{i}^{\prime }\right]}^{1}\right)\right)}^{2}}\right]}^{2},\ldots ,{\left[x_{i}^{n-d}-\frac{\exp\left({\left[X_{i}{\hat{\beta }}_{i}^{\prime }\right]}^{n-d}\right)}{{\left(1+\exp\left({\left[X_{i}{\hat{\beta }}_{i}^{\prime }\right]}^{n-d}\right)\right)}^{2}}\right]}^{2}\right).$$

**Case $x_{i}$ is Poisson** If $x_{i}$ is an independent Poisson random variable with link function $\eta_{i}^{t}=\log\left(\mu_{i}^{t}\right)=\log\left({\left[X_{i}{\hat{\beta }}_{i}^{\prime }\right]}^{t}\right)$, the density is

(20) $$p\left(x_{i}|{\hat{\beta }}_{i},X_{i},\beta_{i}\right)=\prod_{t=d+1}^{n}\frac{\exp{\left({\left[X_{i}{\hat{\beta }}_{i}^{\prime }\right]}^{t}\right)}^{x_{i}^{t}}\exp\left(-\exp\left({\left[X_{i}{\hat{\beta }}_{i}^{\prime }\right]}^{t}\right)\right)}{x_{i}^{t}!}.$$

Then

(21) $$L_{i}=\log\left(p\left(x_{i}|{\hat{\beta }}_{i},X_{i},\gamma_{i}\right)\right)=\sum_{t=d+1}^{n}x_{i}^{t}{\left[X_{i}{\hat{\beta }}_{i}^{\prime }\right]}^{t}-\exp\left({\left[X_{i}{\hat{\beta }}_{i}^{\prime }\right]}^{t}\right)-\log\left(x_{i}^{t}!\right)$$

and diagonal matrix

(22) $$W_{i}:=\mathrm{diag}\left(\exp{\left(X_{i}{\hat{\beta }}_{i}^{\prime }\right)}^{1},\ldots ,\exp{\left(X_{i}{\hat{\beta }}_{i}^{\prime }\right)}^{n-d}\right)$$

for Poisson $x_{i}$ with ${\hat{\phi }}_{i}=1$ and

(23) $$W_{i}:=\mathrm{diag}\left({\left[x_{i}^{d+1}-\exp{\left(X_{i}{\hat{\beta }}_{i}^{\prime }\right)}^{1}\right]}^{2},\ldots ,{\left[x_{i}^{d+(n-d)}-\exp{\left(X_{i}{\hat{\beta }}_{i}^{\prime }\right)}^{n-d}\right]}^{2}\right)$$

for over- or underdispersed Poisson $x_{i}$, i.e., when ${\hat{\phi }}_{i}\neq 1$ and is positive, where t=1,…,n−d.

**Case $x_{i}$ is gamma** If $x_{i}$ is an independent gamma random variable, we consider for the inverse of shape parameter $\kappa_{i}$ for each *t* rate parameter $\kappa_{i}\mu_{i}^{t}$ and for the link function it holds $\mu_{i}^{t}=\frac{1}{\eta_{i}^{t}}=\frac{1}{{\left[X_{i}\beta_{i}\right]}^{t}}.$ For parameters of gamma function $a_{i},b_{i}$ we take $a_{i}=\frac{1}{\kappa_{i}},b_{i}^{t}=\kappa_{i}{\hat{\mu }}_{i}^{t}$ and assume for dispersion ${\hat{\phi }}_{i}=\kappa_{i}$. Then, we have density function

(24) $$p\left(x_{i}|{\hat{\beta }}_{i},\frac{1}{\kappa_{i}},\kappa_{i}{\hat{\mu }}_{i},X_{i},\gamma_{i}\right)=\prod_{t=d+1}^{n}\frac{{\left(x_{i}^{t}\right)}^{\left(\frac{1}{\kappa_{i}}-1\right)}\exp\left(-\frac{x_{i}^{t}}{\kappa_{i}\mu_{i}^{t}}\right)}{{\left(\kappa_{i}\mu_{i}^{t}\right)}^{\frac{1}{\kappa_{i}}}\Gamma \left(\frac{1}{\kappa_{i}}\right)}$$

and log-likelihood $L_{i}=\log\left(p\left(x_{i}|{\hat{\beta }}_{i},\frac{1}{\kappa_{i}},\kappa_{i}{\hat{\mu }}_{i},X_{i},\gamma_{i}\right)\right)$

(25) $$=\sum_{t=d+1}^{n}\left(\left(\frac{1}{\kappa_{i}}-1\right)\log x_{i}^{t}-\frac{x_{i}^{t}}{\kappa_{i}{\hat{\mu }}_{i}^{t}}-\frac{1}{\kappa_{i}}\log\left(\kappa_{i}{\hat{\mu }}_{i}^{t}\right)-\log\Gamma \left(\frac{1}{\kappa_{i}}\right)\right)$$

and diagonal matrix

(26) $$W_{i}:=\mathrm{diag}\left({\left({\hat{\mu }}_{i}^{1}\right)}^{2},\ldots ,{\left({\hat{\mu }}_{i}^{n-d}\right)}^{2}\right)=\mathrm{diag}\left(\frac{1}{{\left({\left[X_{i}{\hat{\beta }}_{i}^{\prime }\right]}^{1}\right)}^{2}},\ldots ,\frac{1}{{\left({\left[X_{i}{\hat{\beta }}_{i}^{\prime }\right]}^{n-d}\right)}^{2}}\right).$$

**Case $x_{i}$ is inverse-Gaussian** If $x_{i}$ is an independent inverse-Gaussian random variable, we consider the inverse of the shape parameter $\xi_{i}$ and link function $\eta_{i}^{t}=\log\left(\mu_{i}^{t}\right)=\log\left({\left[X_{i}{\hat{\beta }}_{i}^{\prime }\right]}^{t}\right)$. Assume dispersion ${\hat{\phi }}_{i}=\xi_{i}$. Then we have density function

(27) $$p\left(x_{i}|{\hat{\beta }}_{i},\xi_{i},{\hat{\mu }}_{i},X_{i},\gamma_{i}\right)=\prod_{t=d+1}^{n}\frac{1}{2\pi \xi_{i}{\left(x_{i}^{t}\right)}^{3}}\exp\left[-\frac{1}{2\xi_{i}}\sum_{t=d+1}^{n}\frac{{\left(x_{i}^{t}-{\hat{\mu }}_{i}^{t}\right)}^{2}}{{\left({\hat{\mu }}_{i}^{t}\right)}^{2}x_{i}^{t}}\right]$$

and log-likelihood $L_{i}=\log\left(p\left(x_{i}|{\hat{\beta }}_{i},\xi_{i}{\hat{\mu }}_{i},X_{i},\gamma_{i}\right)\right)$

(28) $$=\sum_{t=d+1}^{n}\left(-\frac{1}{2\xi_{i}}\sum_{t=d+1}^{n}\frac{{\left(x_{i}^{t}-{\hat{\mu }}_{i}^{t}\right)}^{2}}{{\left({\hat{\mu }}_{i}^{t}\right)}^{2}x_{i}^{t}}-\log\left(2\pi \xi_{i}\right)+3\log\left(x_{i}^{t}\right)\right)$$

and diagonal matrix

(29) $$W_{i}:=\mathrm{diag}\left(\frac{1}{{\hat{\mu }}_{i}^{1}},\ldots ,\frac{1}{\mu_{i}^{n-d}}\right)=\mathrm{diag}\left(\frac{1}{\left({\left[X_{i}{\hat{\beta }}_{i}^{\prime }\right]}^{1}\right)},\ldots ,\frac{1}{\left({\left[X_{i}{\hat{\beta }}_{i}^{\prime }\right]}^{n-d}\right)}\right).$$

One could express similarly $L_{i}$ and $W_{i}$ for other common exponential distributions, applied in GLMs.

### 3.4. Variable Selection by MML in Heterogeneous Graphical Granger Model

For all considered cases of exponential distributions of $x_{i}$ we define the family of models $M\left(\gamma_{i}\right):=\left\{p\left(x_{i}|{\hat{\beta }}_{i},{\hat{\phi }}_{i},X_{i},\gamma_{i}\right),\gamma_{i}\in \Gamma \right\}$ with the corresponding exponential density $p(x_{i}|{\hat{\beta }}_{i},{\hat{\phi }}_{i},X_{i},\gamma_{i})$. First, we present the procedure which for each $x_{i}$ computes the MML code for a set $\gamma_{i}\subset \Gamma$ in Algorithm 1. Then we present Algorithm 2 for computation of ${\hat{\gamma }}_{i}$.
**Algorithm 1** MML Code for $\gamma_{i}$**Input**: $\gamma_{i}\in \Gamma ,d\geq 1$, $|\gamma_{i}|=k_{i}$, series is the matrix of $x_{i}^{t}$, ${\hat{\phi }}_{i}$ dispersion parameter,i=1,…,p,t=1,…,n−d, $\Sigma_{i}$ a unity matrix of size $dk_{i}\;\times \;dk_{i}$, *H* a set of positive numbers;I(γi)=logpki+log(p+1).**Output**: For each *i* minimum of $HMML(x_{i},X_{i},\gamma_{i})$ over *H* is found; **for all**$x_{i}$**do** // Construct the d-lagged matrix $X_{i}$ with time series with indices from $\gamma_{i}$.  // Compute matrix $W_{i}$.  **for all** $\lambda_{i}\in H$ **do**
  // Compute $L_{i}$  // Find the initial estimates of ${\hat{\beta }}_{i}$.   // Compute $MML(x_{i},{\hat{\beta }}_{i},{\hat{\phi }}_{i},\lambda_{i},X_{i},\gamma_{i})$ from (13).  **end for**// to $\lambda_{i}$ // Compute $I\left(x_{i},{\hat{\beta }}_{i},{\hat{\phi }}_{i},{\hat{\lambda }}_{i},X_{i},\gamma_{i}\right)=\min_{\lambda_{i}\in H}MML\left(x_{i},{\hat{\beta }}_{i},{\hat{\phi }}_{i},\lambda_{i},X_{i},\gamma_{i}\right)$.  // $HMML\left(x_{i},X_{i},\gamma_{i}\right):=I\left(x_{i},{\hat{\beta }}_{i},{\hat{\phi }}_{i},{\hat{\lambda }}_{i},X_{i},\gamma_{i}\right)+I\left(\gamma_{i}\right)$. **end for**// to $x_{i}$**return**$HMML(x_{i},X_{i},\gamma_{i})$ for each *i*.

In general, the selection of the best structure $\gamma_{i}$ amounts to evaluate values of $HMML(\gamma_{i})$ for all $\gamma_{i}\subset \Gamma$, i.e., for all $2^{p}$ possible subsets and then to pick the subset with which the minimum of the function was achieved.

### 3.5. Search Algorithms

We will find the best structure of $\gamma_{i}$ with MML code by two approaches. The first way is by the exhaustive search approach exHMML and the second one is by minimizing the HMML by genetic algorithm type procedure called HMMLGA, which we introduce in the following. Since HMML in (12) is a function having multiple local minima, the achievement of the global minimum by these two approaches is not, in general, guaranteed. In [12], a similar genetic algorithm MMLGA was proposed for the Poisson GGM. In this paper, we propose its modification, which is more appropriate for the objective functions that we have here.

The idea of HMMLGA is as follows. Consider an arbitrary $\gamma_{i}\subset \Gamma$ with size $k_{i}$ for a fixed *i*. Define a Boolean vector $Q_{i}$ of length *p* corresponding to a given $\gamma_{i}$, so that it has ones in the positions of the indices of covariates from $\gamma_{i}$, otherwise zeros. Define $HMML\left(Q_{i}\right):=HMML\left(\gamma_{i}\right)$ where $HMML(\gamma_{i})$ is from (12). Genetic algorithm MMLGA executes genetic operations on populations of $Q_{i}$. In the first step, a population of size *m* (*m* an even integer), is generated randomly in the set of all $2^{p}$ binary strings (individuals) of length *p*. Then, we select m/2 individuals in the current population with the lowest value of (12) as the elite subpopulation of parents of the next population. For a predefined number of generated populations $n_{g}$, the crossover operation of parents and the mutation operation of a single parent are executed on the elite to create the rest of the new population. A mutation corresponds to a random change in $Q_{i}$ and a crossover combines the vector entries of a pair of parents. The position of mutation is for each individual selected randomly in contrast to MMLGA, where the position was, for all individuals, the same, and is given as an input parameter. Similarly, the position of crossover in HMMLGA is for each pair of individuals selected randomly. After each run of these two operations on a current population, the current population is replaced with the children with the lowest value of (12) to form the next generation. The algorithm stops after the number of population generations $n_{g}$ is achieved. Since HMML in (12) has multiple local minima, in contract to MMLGA, we selected in the HMMLGA the following strategy: We do not take the first $Q_{i}$ with the sorted HMML values ascendently, but based on the parsimony principle, we take that $Q_{i}$ among all with minimum HMML value, which has the minimum number of ones in $Q_{i}$. Concerning the approach by exhaustive search exHMML, similarly we do not take the first $Q_{i}$ with sorted HMML code ascendently, but also, here, we take that $Q_{i}$, among all with a minimum value of HMML, which has the minimum number of ones in $Q_{i}$. The algorithm HMMLGA is summarized in Algorithm 2.
**Algorithm 2** HMMLGA**Input**: Γ, $d\geq 1,p,n_{g},m$ an even integer;series is the matrix of $x_{i}^{t},i=1,\ldots ,p,t=1,\ldots ,n-d$; **Output**: Adj := adjacency matrix of the output causal graph; // For every $x_{i}$$Q_{i}$ with minimum of (12) is found; **for all**$x_{i}$**do** Create initial population $\left\{Q_{i}^{j},j=1,\ldots ,m\right\}$ at random; Compute HMML(Qij):=I(xi,β^i,ϕ^i,λ^i,Xi,Qij)+pkij+log(p+1) for each j=1,…,m where $k_{i}^{j}$ is the number of ones in $Q_{i}^{j}$; v:=1;  **while** $v\leq n_{g}$ **do**
  u:=1;   **while** u≤m **do**
   Sort $HMML(Q_{i}^{j})$ ascendently and create the elite population; By crossover of $Q_{i}^{j}$ and $Q_{i}^{r}$, r≠j   at a random crossing position create children and add them to elite; Compute $HMML(Q_{i}^{j})$   for each *j*; Mutate a single parent $Q_{i}^{j}$ at a random position; Compute $HMML(Q_{i}^{j})$ for each *j*;   Add the children with minimum $HMML(Q_{i}^{j})$ until the new population is not filled;    u := u + 1;   **end while**// to *u*
  v := v + 1;  **end while**// to *v*
**end for**// to $x_{i}$The *i*-th row of Adj: $Adj_{i}:=Q_{i}$ with min of (12) such that $|Q_{i}|$ is minimum. **return**(Adj)

Our code in Matlab is publicly available at: https://t1p.de/26f3.

#### Computational Complexity of HMMLGA and of exHMML

We used Matlab function *fminsearch* for computation of $HMML(x_{i},{\hat{\beta }}_{i},{\hat{\lambda }}_{i},X_{i},\gamma_{i})$. It is well known that the upper bound of the computational complexity of a genetic algorithm is of order of the product of the size of an individual, of the size of each population, of the number of generated populations and of the complexity of the function to be minimized. Therefore, an upper bound of the computational complexity of HMMLGA for *p* time series, size *p* of an individual, *m* the population size and $n_{g}$ the number of population generations is $O\left(pmn_{g}\right)\times O\left(\mathrm{fminsearch}\right)\;\times \;p$, where O(fminsearch) can also be estimated. The highest complexity in *fminsearch* has the computation of the Hessian matrix, which is the same as for the Fisher information matrix (our matrix $W_{i}$) or the computation of the determinant. The computational complexity of Hessian for *i* fixed for (n−d)×(n−d) matrix is $O(\frac{\left(n-d)(n-d+1\right)}{2})$. An upper bound on the complexity of determinant in (13) is $O({\left(pd\right)}^{3})$ (for proof see e.g., [25]). Denote $M=\max\{{\left(pd\right)}^{3},\frac{\left(n-d)(n-d+1\right)}{2}\}.$ Since we have *p* optimization functions, our upper bound on the computational complexity of HMMLGA is then $O(p^{2}mn_{g}M)$. The computational complexity of exHMML is $p2^{p}O\left(\mathrm{fminsearch}\right)=p2^{p}M$.

## 4. Related Work

In this section, we discuss the related work on the application of two description length based compression schemes for generalized linear models, further the related work on these compression principles applied to causal inference in graphical models, and finally, other papers on causal inference in graphical models for non-Gaussian time series.

Minimum description length (MDL) is another principle based on compression. Similarly as for MML, by viewing statistical modeling as a means of generating descriptions of observed data, the MDL framework (Rissanen [26], Barron et al. [27], and Hansen and Yu [28]) discriminates between competing model classes based on the complexity of each description. The minimum description length principle is based on the idea that one chooses the model that gives the shortest description of data. The methods based on MML and MDL appear mostly equivalent, but there are some differences, especially in interpretation. MML is a Bayesian approach: it assumes that the data-generating process has a given prior distribution. MDL avoids assumptions about the data-generating process. Both methods make use of two-part codes: the first part always represents the information that one is trying to learn, such as the index of a model class (model selection) or parameter values (parameter estimation); the second part is an encoding of the data, given the information in the first part.

Hansen and Yu 2003 in [29] derived objective functions for one-dimensional GLM regression by the minimum description principle. The extension to the multi-dimensional case is however not straighforward. Schmidt and Makalic in [23] used MML87 to derive the MML code of a multivariate GLM ridge regression. Since these works were not designed for time series and do not consider any lag, the mentioned codes cannot be directly used for Granger models.

Marx and Vreeken in [30,31] and Budhathoki and Vreeken [32] applied the MDL principle to the Granger causal inference. The inference in these papers is however done for the bivariate Granger causality and the extension to graphical Granger methods is not straightforward. Hlaváčková-Schindler and Plant in [33] applied both MML and MDL principle to the inference in the graphical Granger models for Gaussian time series. Inference in graphical Granger models for Poisson distributed data using the MML principle was done by the same authors in [12]. To our best knowledge, papers on compression criteria for heterogeneous graphical Granger model have not been published yet.

Among the causal inference on time series, Kim et al. in [7] proposed the statistical framework Granger causality (SFGC) that can operate on point processes, including neural-spike trains. The proposed framework uses multiple statistical hypothesis testing for each pair of involved neurons. A pair-wise hypothesis test was used for each pair of possible connections among all time series and the false discovery rate (FDR) applied. The method can also be used for time series from exponential family.

For a fair comparison with our method, we selected the causal inference methods, which are designed for p≥3 non-Gaussian processes. In our experiments, we used SFGC as a comparison method, and as another comparison method, we selected the method LINGAM from Shimizu et al. [6], which estimates causal structure in Bayesian networks among non-Gaussian time series using structural equation models and independent component analysis. Finally, as a comparison method, we used the HGGM with the adaptive Lasso penalisation method, as introduced in [1] and described in Section 2.2. The experiments reported in the papers with comparison methods were done only in scenarios when the number of time observations is several orders of magnitude greater than the number of time series.

## 5. Experiments

We performed experiments with HMMLGA and with exHMML on processes, which have an exponential distribution of types given in Section 3.3. We used the methods HGGM [1], LINGAM [6] and SFGC [7] for comparison. To assess similarity between the target and output causal graphs in synthetic experiments by all methods, we used the commonly applied *F*-measure, which takes both precision and recall into account.

### 5.1. Implementation and Parameter Setting

The comparison method HGGM uses Matlab package penalized from [34] with adaptive Lasso penalty. The algorithm in this package employs the Fisher scoring algorithm to estimate the regression coefficients. As recommended by the author of penalized in [34] and employed in [1], we used adaptive Lasso with $\lambda_{max}=5$, applying cross validation and taking the best result with respect to *F* measure from the interval $\left(0,\lambda_{max}\right]$. We also followed the recommendation of the authors of LINGAM in [6] and used threshold = 0.05 and the number of boots n/2, where *n* is the length of the time series. In method SFGC , we used the setting recommended by the authors, the significance level 0.05 of FDR.

In HMMLGA and exHMML, the initial estimates of $\beta_{i}$ were achieved by the iteratively re-weighted least square procedure implemented in Matlab function *glmfit*; in the same function, we obtained also the estimates of the dispersion parameters of time series. (Considering initial estimates of $\beta_{i}$ by the IRLS procedure using function *penalized* with ridge penalty gave poor results in the experiments.) In case of gamma distribution, we achieved the estimates of parameters $\kappa_{i}$ by statistical fitting, concretely by Matlab function *gamfit*. The minimization over $\lambda_{i}$ was done by function *fminsearch*, which defined set *H* from Algorithm 1 as positive numbers from interval [0.1, 1000].

### 5.2. Synthetically Generated Processes

To be able to evaluate the performance of HMML and to compare it to other methods, the ground truth, i.e., the target causal graph in the experiments, should be known. In this series of experiments, we examined randomly generated processes, having an exponential distribution of Gaussian and gamma types from Section 3.3, together with the correspondingly generated target causal graphs. The performance of all tested algorithms depends on various parameters, including the number of time series (features), the number of causal relations in Granger causal graph (dependencies), the length of time series, and finally, on the lag parameter. Concerning the calculation of an appropriate lag for each time series; theoretically, it can be done by AIC or BIC. However, the calculation of AIC and BIC assumes that the degrees of freedom are equal to the number of nonzero parameters, which is only known to be true for the Lasso penalty [35], but not known for adaptive Lasso. In our experiments, we followed the recommendation of [1] on how to select the lag of time series in HGGM. It was observed that varying the lag parameter from 3 to 50 did not influence either the performance of HGGM nor SFGC significantly. Based on that, we considered lags 3 and 4 in our experiments.

We examined causal graphs with mixed types of time series for p=5 and p=8 number of features. For each case, we considered causal graphs with higher edge density (dense case) and lower edge density (sparse case), which corresponds to the parameter “dependency” in the code, where the full graph has for *p* time series p(p−1) possible directed edges. Since we concentrate on a short time series in the paper; the length of generated time series varied from 100 to 1000.

#### 5.2.1. Causal Networks with 5 and 8 Time Series

We considered 5 time series with 2 gamma, 2 Gaussian and 1 Poisson distributions, which we generated randomly together with the corresponding network. For the denser case with 5 time series, we generated randomly graphs with 18 edges, and for the sparser case, random graphs with 8 edges. The results of our experiments on causal graphs with 5 features (p=5) are presented in Table 1. Each value in Table 1 represents the mean value of all *F*-measures over 10 random generations of causal graphs for length *n* and lag *d*. For dependency 8, we took strength = 0.9; for dependency 18, we took strength = 0.5 of causal connections.

One can see from Table 1 that HMMLGA and exHMML gave considerably higher precision in terms of F-measure than three other comparison methods, for all considered *n* up to 1000.

In the second network, we considered 8 time series with 7 gamma and 1 Gaussian distributions, which we generated randomly together with a corresponding network. For the denser case, we randomly generated graphs with 52 edges and for the sparser case random graphs with 15 edges. The results are presented in Table 2. Each value in Table 2 represents the mean value of all *F*-measures over 10 random generations of causal graphs for length *n* and lag *d*. For graph with 52 dependencies, we had strength = 0.3; for graph with 15 dependencies, strength = 0.9. Similarly as in the experiments with p=5, one can see in Table 2 for p=8 that both exHMML and HMMLGA gave considerably higher F-measure than the comparison methods for considered *n* up to 1000. The pair-wise hypothesis test used in SFGC for each pair of possible connections among all time series showed its efficiency for long time series in [1,7], however, it was in all experiments in our short-time series scenarios outperformed by LINGAM. The performance of method HGGM, efficient in long-term scenarios [1], was for 5 times series comparable to Lingam; for 8 times, this was the performance of HGGM the weakest from all the methods.

#### 5.2.2. Performance of exHMML and MMLGA

The strategy to select the set $\gamma_{i}$ with minimum HMML and with minimum number of regressors is applied in both methods. In exHMML, all $2^{p}$ possible values of HMML were sorted ascendently. Among those having the same minimum value, that one in the list is selected so that it has minimum number of ones (regressors) and is the last in the list. Similarly, this strategy is applied iteratively in HMMLGA on populations of individuals which have size $m<2^{p}$. This strategy is an improvement with respect to MMLGA [12], where the first $\gamma_{i}$ in the list with minimum MML function was selected. However, since the function HMML has multiple local minima, the convergence to the global minimum by both exHMML and HMMLGA cannot be guaranteed. The different performance of exHMML and HMMLGA for various p and various causal graph density is given by the nature of the objective function in (12) to be minimized. This function has multiple local minima. The above described implementation of both procedures for the exhaustive search and for the genetic algorithm, therefore, without any prior knowledge of the ground truth causal graph, can give different performance of HMMLGA and exHMML. However as shown in the experiments, the achieved local minima are for both methods much closer to the global one than in case of the three rival methods.

### 5.3. Climatological Data

We studied dynamics among seven climatic time series in a time interval. All time series were measured in the station of the Institute for Meteorology of the University of Life Science in Vienna 265 m above sea level [36]. Since weather is a very changeable process, it makes sense to focus on shorter time interval. We considered time series of dewpoint temperature (degree C, dew p), air temperature (degree C, air tmp), relative humidity (%, rel hum), global radiation (W $m^{-2}$, gl rad), wind speed (km/h, w speed), wind direction (degree, w dir), and air pressure (hPa, air pr). All processes were measured every ten minutes, which corresponds to n=432 time observations for each time series. We concentrated on the temporal interactions of these processes during two scenarios. The first one corresponded to 7 to 9 July 2020 which were days with no rain. The second one corresponded to 16 to 18 July 2020 which were rainy days.

Before we applied the methods, we tested the distributions of each time series. In the first scenario (rainy days), air temperature (2) and global radiation (4) followed a gamma distribution and the remaining processes, the dew point temperature (1), relative humidity (3), wind speed (5), wind direction (6), and air pressure (7), following a Gaussian distribution. In the second scenario (dry days), wind direction (6) and air pressure (7) followed a Gaussian distribution, the dew point temperature (1), air temperature (2), relative humidity (3), global radiation (4) and wind speed (5), following a gamma distribution. To decide which of the algorithms exHMML or HMMLGA would be preferable to apply in this real valued experiment, we executed synthetic experiments for constellations of 5 gamma and 2 Gaussian (dry days), as well as of 2 gamma and 5 Gaussian (rainy days), with n=432 for sparse and dense graphs with d=4 and 5, each for 10 random graphs. Higher F-measure was obtained for HMMLGA, therefore we applied the HMMLGA procedure in the climatological experiments.

**Figure 1:**
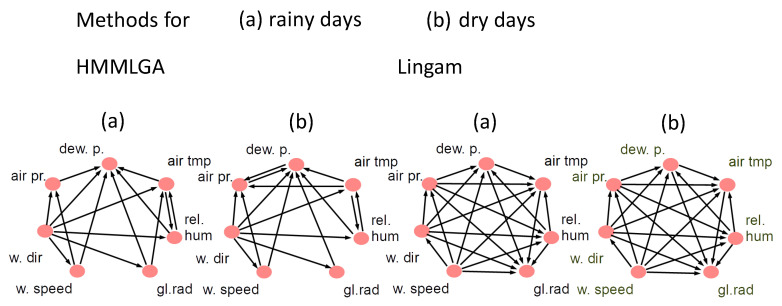
Output causal graphs for method HMMLGA and Lingam for rainy days and dry day scenarios.

**Figure 2:**
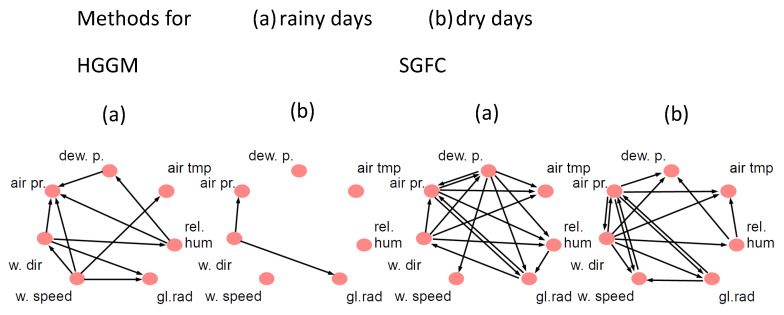
Output causal graphs for method HGGM and SFGC for rainy days and dry day scenarios.

The resulting output graphs for methods HMMLGA, Lingam and HGGM for rainy and dry days gave the same graphs each for both lags; for dry days, we obtained, in HGGM, different graphs for each lag. We were interested in (a) how realistic were the detected temporal interactions of the processes by each method and in each scenario and (b) how realistic were the detected temporal interactions by each method, coming from the difference of graphs for dry and rainy days. In this case, we focused here only on the connections which differed in both graphs for each method. The figures of the output graphs for methods HMMLGA, Lingam, SFGC and HGGM for rainy and dry days can be for lag d=4, seen in Figure 1 and Figure 2.

For Lingam, the ouput graphs for rainy and dry days were identical and complete, so we omitted this method from further analysis.

Based on the expert knowledge [37], the temporal interactions in HMMLGA output graphs in both the rainy and dry scenarios correspond to the reality. In $HMMLGA_{D-R}$, which is the subgraph of HMMLGA of connections of the complement for dry days and of rainy days, the following directed edges in the form (cause, effect) were detected: (air tmp, air pr) and (dew p, air pr). The (direct) influence of dew point on air pressure is more strongly observable during sunny days, since the dew point is not possible to determine during rainy days. Similarly, the causal influence of air temperature on the air pressure is stronger during sunny days than during rainy days. So, both detected edges in HMMLGA were realistic. $HMMLGA_{R-D}$ was empty. Output graph $HGGM_{D-R}$ gave no edges. For $HGGM_{R-D}$, we obtained these directed edges: (dew p, air pr), which is, during rain, not observable, but the achieved influence (rel hum, dew p) is also during rain observable. Moreover, (rel hum, air pr) are observable (as humidity increases, pressure decreases). The edge (w speed, w dir) is not observable in reality, (w speed, air pr) is observable (higher wind speeds will show lower air pressure); also (w speed, air tmp) and (w speed, gl rad) are observable, however direct effect (w dir, rel hum) is not observable in reality. So, $HGGM_{R-D}$ had 2 falsely detected directions out of 8. Graph $SFGC_{R-D}$ gave this edge (dew p, air pr). Similarly, as in the case of HGGM, this edge is, during rain, not observable; (dew p, air tmp)—is during rain not observable; (dew p, w speed)—is during rain not observable; (dew p, rel hum)—is during rain not observable; (dew p, gl rad)—is during rain not observable; (rel hum, gl rad)—is during rain observable; (gl rad, w speed)—is during rain not observable; (gl rad, w dir)—is during rain not observable. So, $SFGC_{R-D}$ had 7 falsely detected directions out of 8. The output of $SFGC_{D-R}$ gave these edges: (rel hum, dew p)—this is during a dry period observable; (rel hum, air tmp)—this is during a dry period observable; (gl rad, w speed)—this is during a dry period observable; (dev p, air tmp)—this is during a dry period observable; (air press, w dir)—this is during a dry period observable; (w speed, air pr)—this is during a dry period observable; (air pr, w speed) is during dry period in reality observable. So, $SFGC_{D-R}$ had 7 correctly detected directions out of 7.

We conclude that, in this climatological experiment, method HMMLGA, followed by SFGC, gave the most realistic causal connections with respect to the comparison methods.

### 5.4. Electrohysterogram Time Series

In the current obstetrics, there is no effective way of preventing preterm birth. The main reason is that no good objective method is known to evaluate the stepwise progression of pregnancy through to labor [38]. Any better understanding of the underlying labor dynamics can contribute to prevent preterm birth, which is the main cause of mortality in newborns. External recordings of the electrohysterogram (EHG) can provide new knowledge on uterine electrical activity associated with contractions.

**Figure 3:**
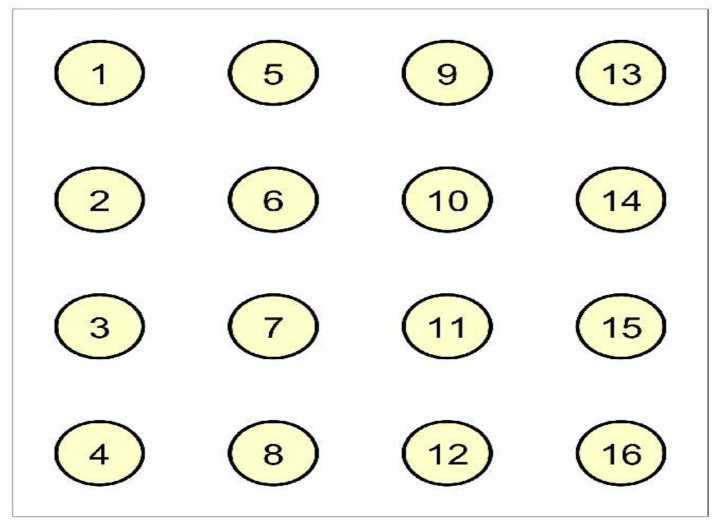
The ordering of the electrodes as mounted on the abdomen of women.

We considered a database of 4-by-4 electrode EHG recordings performed on pregnant women, which were recorded in Iceland between 2008 and 2010 and are available via PhysioNet (PhysioBank ATM) [39]. This EHC grid (in the matrix form) was placed on the abdomen of the pregnant women. The electrode numbering, as considered in [38], can be found in Figure 3.

We applied the recordings, concretely for EHG signal for women in the third phase of pregnancy and during labor, to all the methods. We selected all (five) mothers for which the recordings were performed, both in the third trimester and during labor. Since there is no ground truth known on how the dynamics among the electrodes should look like for both modalities, we set a modest objective for us, whether HMMLGA and the comparison methods are able to distinguish labor from pregnancy from the EHG recordings. During labor, a higher density of interactions among electrodes is expected than during pregnancy, due to the higher occurrence of contractions of the uterine smooth muscles, which is also supported by some recent research in obstetrics, e.g., [40].

The 16 electromyographic time series (channels) were taken for all women (woman 11, 27, 30, 31 and 34), for each in the third trimester (P) and during labor (L). The observations in time series correspond to the time resolution every 5th microsecond. The time series in the databasis are commented by information about contraction, possible contraction, participant movement, participant change of position, fetal movement and equipment manipulation. By statistical fitting, we found out that all 16 time series followed Poisson distribution (setting raw ADC units in the Physionet database). We analysed the causal connections of each method for labor and pregnancy for all five women.

**Figure 4:**
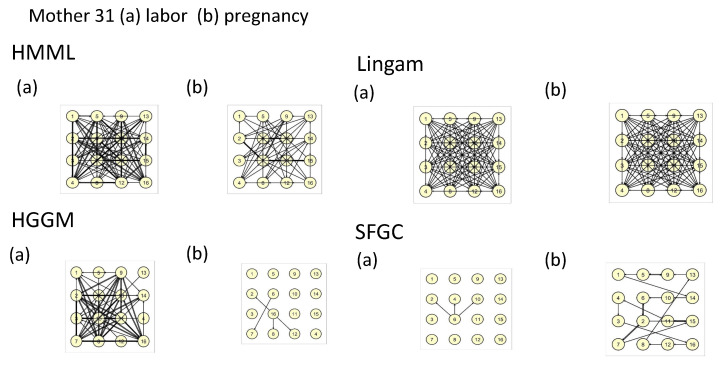
Output causal graphs for mother 31 during (**a**) labor and (**b**) pregnancy for all methods.

Since HMMLGA had higher F-measure than exHMML in the synthetic experiments with 16 Poisson time series, we considered further only HMMLGA in this real data experiment. In the synthetic experiments in [12], Poisson time series showed the highest F-measure on short time series, i.e., the case when the number of time observations is smaller than approximately two orders times the number of time series. Based on this, we took the last 1200 observations for labor, since in the last phase, it was sure the labor had already started and the contractions had increased in time. Labor still continued for another few hours after the EHG recording finished for each of five women. For pregnancy time series, we took also 1200 observations, starting the moment where all electrodes had been fixed. The hypothesis, that during labor all electrodes were activated was confirmed by HMMLGA, HGGM and Lingam at all mothers. The hypothesis, that the causal graph during labor had higher density of causal connections than in the pregnancy case, was confirmed at all mothers by HMMLGA, for HGGM for mothers 30 and 31, but for SFGC and Lingam, we could not confirm it. In fact, Lingam gave identical complete causal graphs for both labor and pregnancy cases. The real computational time for Lingam (with 100 boots, as recommended by the authors) was for 16 time series and both labor and pregnancy modalities cca 12 h (in HP Elite Notebook); on the other side, for other methods, the time was in order of minutes. We present the causal graphs of all methods for labor and pregnant phase of mother 31 in Figure 4.

One can see that the density of connections by HMMLGA for labor is higher than for pregnancy. Causal graphs of HMMLGA for all mothers were for labor also denser than the pregnancy one. To make some more concrete hypotheses about the temporal interactions among the electrodes based on contractions, we would probably have to consider only intervals about which we know that they are without or with a limited number of artifacts in terms of participant movement, participant change of position, etc.

## 6. Conclusions

Common graphical Granger models in scenarios with short time series suffer often from overestimation, including the heterogeneous graphical Granger model. To remedy this, in this paper, we proposed to use the minimum message length principle for determination of causal connections in the heterogeneous graphical Granger model. Based on the dispersion coefficient of the target time series and on the initial maximum likelihood estimates of the regression coefficients, we proposed a minimum message length criterion to select the subset of causally connected time series with each target time series, and we derived its concrete form for various exponential distributions. We found this subset by a genetic-type algorithm (HMMLGA), which we have proposed as well as by exhaustive search (exHMML). We evaluated the complexity of these algorithms. The code in Matlab is provided. We demonstrated superiority of both methods with respect to the comparison methods in synthetic experiments in short data scenarios. In two real data experiments, the interpretation of the causal connections as the result of HMMLGA was the most realistic with respect to the comparison methods. The superiority of HMMLGA with respect to the comparison methods for short time series can be explained by utilizing the dispersion of time series in the criterion as an additional (prior) information, as well as the fact that this criterion is optimized in the finite search space. 

## Figures and Tables

**Table 1:** p=5, average *F*-measure for each method, HMML, $n_{g}=10$, m=20, HGGM with $\lambda_{max}=5$, LINGAM with n/2 boots. The first one subtable is for d=3, the second one for d=4.

dense g. 18, n=	100	300	500	1000;	sparse g. 8, n=	100	300	500	1000
**exHMML**	0.69	0.83	0.82	0.88	**exHMML**	0.70	0.72	0.72	**0.67**
**HMMLGA**	**0.73**	**0.90**	**0.89**	**0.90**	**HMMLGA**	**0.73**	**0.76**	**0.74**	**0.67**
HGGM	0.5	0.48	0.54	0.52	HGGM	0.52	0.36	0.66	0.36
LINGAM	0.57	0.58	0.62	0.58	LINGAM	0.58	0.54	0.69	0.45
SFGC	0.33	0.26	0.26	0.33	SFGC	0.14	0.35	0.44	0.31
dense g. 18, n=	100	300	500	1000;	sparse g. 8, n=	100	300	500	1000
**exHMML**	0.71	0.73	0.83	0.83	**exHMML**	**0.67**	**0.80**	**0.80**	0.68
**HMMLGA**	**0.82**	**0.79**	**0.87**	**0.92**	**HMMLGA**	**0.67**	0.73	0.77	**0.70**
HGGM	0.44	0.37	0.40	0.39	HGGM	0.53	0.47	0.65	0.36
LINGAM	0.71	0.58	0.58	0.65	LINGAM	0.33	0.52	0.74	0.46
SFGC	0.43	0.55	0.42	0.63	SFGC	0.35	0.59	0.42	0.38

**Table 2:** p=8, average *F*-measure for each method, HMML, with d=3, $n_{g}=10$, m=20, HGGM with $\lambda_{max}=5$, LINGAM with n/2 boots. The first subtable is for d=3, the second one for d=4.

dense g. 52, n=	100	300	500	1000;	sparse g. 15, n=	100	300	500	1000
**exHMML**	0.68	**0.78**	**0.79**	0.82	**exHMML**	**0.69**	**0.73**	**0.77**	**0.64**
**HMMLGA**	**0.84**	0.67	0.66	**0.87**	**HMMLGA**	0.57	0.69	0.7	0.56
HGGM	0.16	0.17	0.17	0.17	HGGM	0.2	0.09	0.18	0.17
LINGAM	0.62	0.54	0.51	0.55	LINGAM	0.28	0.33	0.4	0.19
SFGC	0.32	0.21	0.35	0.20	SFGC	0.3	0.24	0.22	0.19
dense g. 52, n=	100	300	500	1000;	sparse g. 15, n=	100	300	500	1000
**exHMML**	0.59	0.64	0.56	0.75	**exHMML**	**0.58**	**0.84**	**0.80**	**0.69**
**HGGMGA**	**0.77**	**0.72**	**0.63**	**0.79**	**HMMLGA**	0.42	0.69	0.70	0.56
HGGM	0.16	0.16	0.18	0.17	HGGM	0.17	0.10	0.18	0.19
LINGAM	0.62	0.54	0.51	0.55	LINGAM	0.27	0.33	0.40	0.18
SFGC	0.36	0.45	0.82	0.83	SFGC	0.29	0.29	0.24	0.20

## Appendix A. Derivation of the MML Criterion for HGGM

Assume *p* independent random variables from the exponential family which are represented by time series $x_{i}^{t},t=d+1,\ldots ,n$ and for each *i* be ${\hat{\phi }}_{i}$ the given estimate of its dispersion. Consider the problem (10) for a given lag d>0.

We consider now $\gamma_{i}$ fixed, so for simplicity of writing we omit it from the list of variables of the functions. Having function $L_{i}$, we can now compute an initial estimate of ${\hat{\beta }}_{i}$ from (10) which is the solution to the system of score equations. Since $L_{i}$ forms a convex function, one can use standard convex optimization techniques (e.g., Newton-Raphson method) to solve these equations numerically. (In our code, we use the Matlab implementation of an iteratively reweighted least squares (IRLS) algorithm of the Newton–Raphson method). Assume now we have an initial solution ${\hat{\beta }}_{i}$ from (10).

Having parameters ${\hat{\beta }}_{i}$, ${\hat{\phi }}_{i}$, $\Sigma_{i}$$W_{i}$ and $\lambda_{i}$, we need to construct the function $HMML(\gamma_{i})$: We use for each i=1,…,p and for regression (10) Formula (18) from [23] i.e., the case when we plug in variables α:=0 and $\beta :={\hat{\beta }}_{i}$ and $X:=X_{i}$, $y:=x_{i}$, n:=n−d, $k:=k_{i}$, $\theta :={\hat{\beta }}_{i}$, $\lambda :=\lambda_{i}$, $\phi :={\hat{\phi }}_{i}$, $S=\Sigma_{i}$ be the unity matrix of dimension $dk_{i}$. The corrected Fisher information matrix for the parameters $\beta_{i}$ is then $J\left({\hat{\beta }}_{i}|{\hat{\phi }}_{i},\lambda_{i}\right)=\left(\frac{1}{\phi_{i}}\right)X_{i}^{\prime }W_{i}X_{i}+\lambda_{i}\Sigma_{i}$. Function c(m) for $m:=k_{i}+1$ is then $c\left(k_{i}+1\right)=-\frac{k_{i}+1}{2}\log\left(2\pi \right)+\frac{1}{2}\log\left(\left(k_{i}+1\right)\pi \right)-0.5772$ and the constants which are independent of $k_{i}$ we omitted from the HMML code, since the optimization w.r.t. $\gamma_{i}$ is independent of them. Among all subsets $\gamma_{i}\in \Gamma$, there are pki subsets of size $k_{i}$. If nothing is known a priori about the likelihood of any covariate $x_{i}$ being included in the final model, a prior that treats all subset sizes equally likely $\pi (|\gamma_{i}|)=1/\left(p+1\right)$ is appropriate [23]. This gives the code length I(γi)=logpki+log(p+1) as in (12).

## Appendix B. Derivation of *L_i_*, **W***_i_*, *ϕ_i_* for Various Exponential Distributions of x *_i_*

**Case $x_{i}$ is Gaussian** Since in this case is $\phi_{i}=\sigma_{i}^{2}$ its variance, we will omit $\phi_{i}$ from the list of parameters which condition function *p*. $L_{i}$ in (15) is obtained directly from (14) by applying logarithm on it. By plugging values for identity link corresponding to the Gaussian case as $\eta_{i}^{t}=\mu_{i}^{t}={\left[X_{i}\beta_{i}\right]}^{t}$ and $\frac{\delta \eta_{i}^{t}}{\delta \mu_{i}^{t}}=1$ into Formula (13) from [23], matrix $W_{i}=I_{k_{i}d\times k_{i}d}$ is directly obtained.

**Case $x_{i}$ is binomial** Assuming $\phi_{i}$ be a constant, we can omit $\phi_{i}$ from the list of parameters which condition function *p*. $L_{i}$ in (17) is obtained directly from (16) by applying logarithm on it. As in the previous case, it is obtained by plugging values into formula (13) from [23]. Value of $W_{i}$ from (19) is obtained by plugging values for logit link corresponding to the binomial case as $\eta_{i}^{t}={\left[X_{i}\beta_{i}\right]}^{t}=\log\left(\frac{\mu_{i}^{t}}{1-\mu_{i}^{t}}\right)$ and $\frac{\delta \eta_{i}^{t}}{\delta \mu_{i}^{t}}=\frac{1}{\mu_{i}^{t}\left(1-\mu_{i}^{t}\right)}$ into Formula (13) from [23]. In case we cannot assume $\phi_{i}=1$, we apply the sandwich estimate of the covariance matrix of ${\hat{\beta }}_{i}$ for robust estimation which for a general logistic regression can be found in e.g., [41]) and in our case it gives matrix $W_{i}$ in the form $W_{i}=\mathrm{diag}\left({\left(x_{i}^{1}-\frac{\exp\left({\left[X_{i}{\hat{\beta }}_{i}^{\prime }\right]}^{1}\right)}{{\left(1+\exp\left({\left[X_{i}{\hat{\beta }}_{i}^{\prime }\right]}^{1}\right)\right)}^{2}}\right)}^{2},\ldots ,{\left(x_{i}^{n-d}-\frac{\exp\left({\left[X_{i}{\hat{\beta }}_{i}^{\prime }\right]}^{n-d}\right)}{{\left(1+\exp\left({\left[X_{i}{\hat{\beta }}_{i}^{\prime }\right]}^{n-d}\right)\right)}^{2}}\right)}^{2}\right)$.

**Case $x_{i}$ is Poisson** First we will express the log-likelihood function $L_{i}$ in terms of parameters $\beta_{i}$. Since we use Poisson model for $x_{i}$ having the Poisson distribution or overdispersed Poisson, we omit $\phi_{i}$ from the list of parameters which condition function *p*. For a given set of parameters $\beta_{i}$, the probability of attaining $x_{i}^{d+1},\ldots ,x_{i}^{n}$ is given by $p(x_{i}^{d+1},\ldots ,x_{i}^{n}|X_{i},\beta_{i})$ $=\prod_{t=d+1}^{n}\frac{{\left(\mu_{i}^{t}\right)}^{x_{i}^{t}}\exp\left(-\mu_{i}^{t}\right)}{(x_{i}^{t})!}=\prod_{t=d+1}^{n}\frac{\exp{\left({\left[X_{i}\beta_{i}^{\prime }\right]}^{t}\right)}^{x_{i}^{t}}\exp\left(-\exp\left({\left[X_{i}\beta_{i}^{\prime }\right]}^{t}\right)\right)}{x_{i}^{t}!}$ and $\eta_{i}^{t}=\exp\left({\left[X_{i}\beta_{i}^{\prime }\right]}^{t}\right)$, (recalling the notation from Section 3.2, ${\left[X_{i}\beta_{i}^{\prime }\right]}^{t}$ denotes the t-th coordinate of the vector $X_{i}\beta_{i}^{\prime }$). The log-likelihood in terms of $\beta_{i}$ is $L_{i}=\log p\left(\beta_{i}|x_{i},X_{i}\right)=\sum_{t=d+1}^{n}x_{i}^{t}{\left[X_{i}\beta_{i}^{\prime }\right]}^{t}-\exp\left({\left[X_{i}\beta_{i}^{\prime }\right]}^{t}\right)-\log\left(x_{i}^{t}!\right).$ Now we derive matrix $W_{i}$ for $x_{i}$ with (exact) Poisson distribution: The Fisher information matrix $J_{i}=J\left(\beta_{i}\right)=-E_{\beta_{i}}\left(\nabla^{2}L_{i}\left(\beta_{i}|x_{i},X_{i}\right)\right)$ may be obtained by computing the second order partial derivatives of $L_{i}$ for $r,s=1,\ldots ,k_{i}$. This gives

(A1) $$\begin{array}{ccc} \frac{\delta^{2}L_{i}\left(\beta_{i}|x_{i},X_{i}\right)}{\delta^{2}\beta_{i}^{r}\beta_{i}^{s}} & = & \frac{\delta L_{i}}{\delta \beta_{i}^{s}}\sum_{t=d+1}^{n}\left[x_{i}^{t}\sum_{l=1}^{d}x_{r}^{t-l}-\exp\left(\sum_{j=1}^{k_{i}}\sum_{l=1}^{d}x_{j}^{t-l}\beta_{j}^{l}\right)\sum_{l=1}^{d}x_{r}^{t-l}\right]\\ \; & = & -\sum_{t=d+1}^{n}\exp\left(\sum_{j=1}^{k_{i}}\sum_{l=1}^{d}x_{j}^{t-l}\beta_{j}^{l}\right)\left(\sum_{l=1}^{d}x_{s}^{t-l}\right)\left(\sum_{l=1}^{d}x_{r}^{t-l}\right). \end{array}$$

If we denote $W_{i}:=diag\left(\exp\left(\sum_{j=1}^{k_{i}}\sum_{l=1}^{d}x_{j}^{d+1-l}\beta_{j}^{l}\right),\ldots ,\exp\left(\sum_{j=1}^{k_{i}}\sum_{l=1}^{d}x_{j}^{n-l}\beta_{j}^{l}\right)\right)$ then we have Fisher information matrix $J\left(\beta_{i}\right)={\left(X_{i}\right)}^{\prime }W_{i}X_{i}$. Alternatively, $W_{i}$ can be obtained by plugging values into formula (13) from [23]. Value of $W_{i}$ from (22) is obtained by plugging values for log link corresponding to the Poisson case as $\eta_{i}^{t}={\left[X_{i}\beta_{i}\right]}^{t}=\log\left(\mu_{i}^{t}\right)$ and $\frac{\delta \eta_{i}^{t}}{\delta \mu_{i}^{t}}=\frac{1}{\mu_{i}^{t}}$ into Formula (13) from [23].

Derivation of matrix $W_{i}$ for $x_{i}$ with overdispersed Poisson distribution: Assume now the dispersion parameter $\phi_{i}>0,\neq 1$. The variance of the overdispersed Poisson distribution is $\phi_{i}\mu_{i}$. We know that the Poisson regression model can be still used in overdispersed settings and the function $L_{i}$ is the same as $L_{i}\left(\beta_{i}\right)$ derived above. We use the robust sandwich estimate of covariance of ${\hat{\beta }}_{i}$ as it was proposed in [42] for general Poisson regression. The Fisher information matrix of overdispersed problem is $J_{i}=J\left(\beta_{i}\right)={\left(X_{i}\right)}^{\prime }W_{i}X_{i}$ where $W_{i}$ is constructed for $x_{i}$ Poisson based on [42] and has the form $W_{i}=$ $\mathrm{diag}([x_{i}^{d+1}-\exp\left(\sum_{j=1}^{k_{i}}\sum_{l=1}^{d}x_{j}^{d+1-l}\beta_{j}^{l}\right){]}^{2},\ldots ,\left[x_{i}^{n}-\exp\left(\sum_{j=1}^{k_{i}}\sum_{l=1}^{d}x_{j}^{n-l}\beta_{j}^{l}\right){]}^{2}\right)$.

**Case $x_{i}$ is gamma**$L_{i}$ in (25) is obtained directly from (24) by applying logarithm on it. By plugging values for log link corresponding to the gamma case as $\eta_{i}^{t}=\frac{1}{\mu_{i}^{t}}$ and $\frac{\delta \eta_{i}^{t}}{\delta \mu_{i}^{t}}=\frac{1}{{\left(\mu_{i}^{t}\right)}^{2}}$ into Formula (13) from [23], matrix $W_{i}$ from (26) is directly obtained.

**Case $x_{i}$ is inverse-Gaussian**$L_{i}$ in (28) is obtained directly from (24) by applying logarithm on it. By plugging values for log link corresponding to the inverse-Gaussian case as $\eta_{i}^{t}={\left[X_{i}\beta_{i}\right]}^{t}=\log\left(\mu_{i}^{t}\right)$ and $\frac{\delta \eta_{i}^{t}}{\delta \mu_{i}^{t}}=\frac{1}{\mu_{i}^{t}}$ into Formula (13) from [23], matrix $W_{i}$ from (29) is directly obtained.

## References

[1] Behzadi S., Hlaváčková-Schindler K., Plant C. (2019). Granger Causality for Heterogeneous Processes. Pacific-Asia Conference on Knowledge Discovery and Data Mining.

[2] Zou H. (2006). The adaptive lasso and its oracle property. J. Am. Stat. Assoc..

[3] Hryniewicz O., Kaczmarek K. (2015). Forecasting short time series with the bayesian autoregression and the soft computing prior information. Strengthening Links Between Data Analysis and Soft Computing.

[4] Bréhélin L. (2006). A Bayesian approach for the clustering of short time series. Rev. D’Intell. Artif..

[5] Wallace C.S., Boulton D.M. (1968). An information measure for classification. Comput. J..

[6] Shimizu S., Inazumi T., Sogawa Y., Hyvärinen A., Kawahara Y., Washio T., Hoyer P.O., Bollen K. (2011). DirectLiNGAM: A direct method for learning a linear non-Gaussian structural equation model. J. Mach. Learn. Res..

[7] Kim S., Putrino D., Ghosh S., Brown E.N. (2011). A Granger causality measure for point process models of ensemble neural spiking activity. PLoS Comput. Biol..

[8] Arnold A., Liu Y., Abe N. Temporal causal modeling with graphical Granger methods. Proceedings of the 13th ACM SIGKDD International Conference on Knowledge Discovery and Data Mining.

[9] Shojaie A., Michailidis G. (2010). Discovering graphical Granger causality using the truncating lasso penalty. Bioinformatics.

[10] Lozano A.C., Abe N., Liu Y., Rosset S. Grouped graphical Granger modeling methods for temporal causal modeling. Proceedings of the 15th ACM SIGKDD International Conference on Knowledge Discovery and Data Mining.

[11] Nelder J., Wedderburn R. (1972). Generalized Linear Models. J. R. Stat. Soc. Ser. A (General).

[12] Hlaváčková-Schindler K., Plant C. Poisson Graphical Granger Causality by Minimum Message Length. Proceedings of the European Conference on Machine Learning and Principles and Practice of Knowledge Discovery in Databases 2020 (ECML/PKDD).

[13] Granger C.W. (1969). Investigating causal relations by econometric models and cross-spectral methods. Econometrica.

[14] Mannino M., Bressler S.L. (2015). Foundational perspectives on causality in large-scale brain networks. Phys. Life Rev..

[15] Maziarz M. (2015). A review of the Granger-causality fallacy. J. Philos. Econ. Reflect. Econ. Soc. Issues.

[16] Granger C.W. (1988). Some recent development in a concept of causality. J. Econom..

[17] Lindquist M.A., Sobel M.E. (2011). Graphical models, potential outcomes and causal inference: Comment on Ramsey, Spirtes and Glymour. NeuroImage.

[18] Spirtes P., Glymour C.N., Scheines R., Heckerman D. (2000). Causation, Prediction, and Search.

[19] Glymour C. (2013). Counterfactuals, graphical causal models and potential outcomes: Response to Lindquist and Sobel. NeuroImage.

[20] Marinescu I.E., Lawlor P.N., Kording K.P. (2018). Quasi-experimental causality in neuroscience and behavioural research. Nat. Hum. Behav..

[21] Wallace C.S., Freeman P.R. (1987). Estimation and inference by compact coding. J. R. Stat. Soc. Ser. B.

[22] Wallace C.S., Dowe D.L. (1999). Minimum message length and Kolmogorov complexity. Comput. J..

[23] Schmidt D.F., Makalic E. (2013). Minimum message length ridge regression for generalized linear models. Australasian Joint Conference on Artificial Intelligence.

[24] Segerstedt B. (1992). On ordinary ridge regression in generalized linear models. Commun. Stat. Theory Methods.

[25] Computational Complexity of Mathmatical Operations. https://en.wikipedia.org/wiki/Computational_complexity_of_mathematical_operations.

[26] Rissanen J. (1989). Stochastic Complexity in Statistical Inquiry.

[27] Barron A., Rissanen J., Yu B. (1998). The minimum description length principle in coding and modeling. IEEE Trans. Inf. Theory.

[28] Hansen M., Yu B. (2001). Model selection and minimum description length principle. J. Am. Stat. Assoc..

[29] Hansen M.H., Yu B. (2003). Minimum description length model selection criteria for generalized linear models. Lect. Notes Monogr. Ser..

[30] Marx A., Vreeken J. Telling cause from effect using MDL-based local and global regression. Proceedings of the 2017 IEEE International Conference on Data Mining.

[31] Marx A., Vreeken J. Causal inference on multivariate and mixed-type data. Proceedings of the Joint European Conference on Machine Learning and Knowledge Discovery in Databases.

[32] Budhathoki K., Vreeken J. (2018). Origo: Causal inference by compression. Knowl. Inf. Syst..

[33] Hlaváčková-Schindler K., Plant C. Graphical Granger causality by information-theoretic criteria. Proceedings of the European Conference on Artificial Intelligence 2020 (ECAI).

[34] McIlhagga W.H. (2016). Penalized: A MATLAB toolbox for fitting generalized linear models with penalties. J. Stat. Softw..

[35] Zou H., Hastie T., Tibshirani R. (2007). On the “degrees of freedom” of the lasso. Ann. Stat..

[36] https://meteo.boku.ac.at/wetter/mon-archiv/2020/202009/202009.html.

[37] Zentralanstalt für Meteorologie und Geodynamik 1190 Vienna, Hohe Warte 38. https://www.zamg.ac.at/cms/de/aktuell.

[38] Alexandersson A., Steingrimsdottir T., Terrien J., Marque C., Karlsson B. (2015). The Icelandic 16-electrode electrohysterogram database. Nat. Sci. Data.

[39] https://www.physionet.org.

[40] Mikkelsen E., Johansen P., Fuglsang-Frederiksen A., Uldbjerg N. (2013). Electrohysterography of labor contractions: Propagation velocity and direction. Acta Obstet. Gynecol. Scand..

[41] Agresti A. (2003). Categorical Data Analysis.

[42] Huber P.J. (1967). The behavior of maximum likelihood estimates under nonstandard conditions. Proceedings of the Fifth Berkeley Symposium on Mathematical Statistics and Probability.

